# The prevalence and predictors of cardiovascular diseases in Kherameh cohort study: a population-based study on 10,663 people in southern Iran

**DOI:** 10.1186/s12872-022-02683-w

**Published:** 2022-05-28

**Authors:** Najibullah Baeradeh, Masoumeh Ghoddusi Johari, Leila Moftakhar, Ramin Rezaeianzadeh, Seyed Vahid Hosseini, Abbas Rezaianzadeh

**Affiliations:** 1 Student Research Committee, Shiraz University of Medical Sciences, Shiraz, Iran; 2 Breast Diseases Research Center, Shiraz University of Medical Sciences, Shiraz, Iran; 3 Colorectal Research Center, Shiraz University of Medical Sciences, Shiraz, Iran; 4 Non-Communicable Diseases Research Center, Shiraz University of Medical Sciences, Shiraz, Iran; 5 Experimental Medicine Program, Department of Medicine, Faculty of Medicine, University of British Columbia, Vancouver, British Columbia Canada

**Keywords:** Cardiovascular disease, Predictors, Prevalence, Kherameh cohort

## Abstract

**Background:**

The prevalence of cardiovascular disease (CVD) is rapidly increasing in the world. The present study aimed to assess the prevalence and Predictors factors of CVD based on the data of Kherameh cohort study.

**Methods:**

The present cross-sectional, analytical study was done based on the data of Kherameh cohort study, as a branch of the Prospective Epidemiological Studies in Iran (PERSIAN). The participants consisted of 10,663 people aged 40–70 years. CVD was defined as suffering from ischemic heart diseases including heart failure, angina, and myocardial infarction. Logistic regression was used to model and predict the factors related to CVD. Additionally, the age-standardized prevalence rate (ASPR) of CVD was determined using the standard Asian population.

**Results:**

The ASPR of CVD was 10.39% in males (95% CI 10.2–10.6%) and 10.21% in females (95% CI 9.9–10.4%). The prevalence of CVD was higher among the individuals with high blood pressure (58.3%, *p* < 0.001) as well as among those who smoked (28.3%, *p* = 0.018), used opium (18.2%, *p* = 0.039), had high triglyceride levels (31.6%, *p* = 0.011), were overweight and obese (66.2%, *p* < 0.001), were unmarried (83.9%, *p* < 0.001), were illiterate (64.2%, *p* < 0.001), were unemployed (60.9%, *p* < 0.001), and suffered from diabetes mellitus (28.1%, *p* < 0.001). The results of multivariable logistic regression analysis showed that the odds of having CVD was 2.25 times higher among the individuals aged 50–60 years compared to those aged 40–50 years, 1.66 folds higher in opium users than in non-opium users, 1.37 times higher in smokers compared to non-smokers, 2.03 folds higher in regular users of sleeping pills than in non-consumers, and 4.02 times higher in hypertensive individuals than in normotensive ones.

**Conclusion:**

The prevalence of CVD was found to be relatively higher in Kherameh (southern Iran) compared to other places. Moreover, old age, obesity, taking sleeping pills, hypertension, drug use, and chronic obstructive pulmonary disease had the highest odds ratios of CVD.

## Introduction

In recent decades, the rapid growth of Non-Communicable Diseases (NCDs) has become a serious health challenge threatening the health and economic development of communities [1]. Chronic NCDs such as Cardiovascular Disease (CVD), cancer, respiratory diseases, and Diabetes Mellitus (DM) are the leading causes of death worldwide [2]. The incidence of CVD is rapidly increasing in the world and, consequently, it is currently considered the leading cause of death in both developing and developed countries [3, 4]. CVD was responsible for the death of 17.9 million people worldwide in 2016, accounting for 31% of all global deaths [5]. Additionally, the prevalence of CVD increased from 257 million in 1990 to 550 million in 2019. The number of associated deaths showed a steady increase, as well [6]. Evidence has also indicated the global prevalence of angina to be 0.73–14.4% in females and 0.76–15.1% amongst males [7]. Nonetheless, burden, mortality, and prevalence of CVD vary in different parts of the world [8]. Over the past 20 years, the incidence of CVD has witnessed a significant decrease in industrialized countries and an increase in developing countries including the Eastern Mediterranean region [9, 10]. As an Eastern Mediterranean country, Iran has adopted a Western lifestyle. Such changes, along with improved health services, have led to the improvement of life expectancy as well as an increase in the prevalence of NCDs including CVD [11, 12]. In Iran, as in other parts of the world, CVD is the leading cause of death and its prevalence has been estimated as 9.2% in Sari located in north of the country [13, 14]. Nevertheless, contradictory results have been obtained regarding the prevalence of CVD based on gender in different studies. Some studies have reported a higher prevalence in males, while some others have found it to be higher among females [15–18]. According to what was mentioned above, CVD is the most important cause of death in the world as well as in Iran, and the prevalence of this disease and its risk factors is varied in different regions. In recent decades, population-based cohort studies have provided the ground for the performance of accurate cross-sectional studies in this field. The present study aims to determine the prevalence and prognostic factors of CVD based on the data of Kherameh cohort study to provide a more comprehensive image of the disease burden, which will in turn result in the provision of more efficient and targeted health services.

## Methods

### Study design and population

**Fig. 1:**
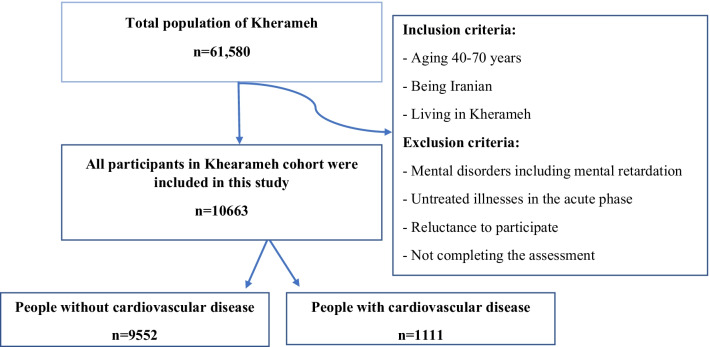
Flow chart of the participants’ enrolment into the research

The present cross-sectional, analytical study was done based on the data of Kherameh cohort study, as a branch of Prospective Epidemiological Studies in Iran (PERSIAN). The Persian Cohort Study was launched in 18 separate geographical areas in Iran in 2014 and included all major Iranian ethnic groups. It is one of the largest cohort studies in the region and its rationale, goals, and design have already been published [19]. Kherameh is one of the southern cities of Fars province with a population of 61,580 people. Kherameh cohort is one of the branches of Fars cohort that was started with a population of 10,663 people in the 40–70 age group in 2014 to determine the prevalence and risk factors of NCDs at baseline and during the follow-up period. All participants of Kherameh cohort study were entered into the present research through census (Fig. 1).

## Measurement

### Demographic and lifestyle information

The present findings were extracted from a research project approved by the Ethics Committee of Shiraz University of Medical Sciences (code: IR.SUMS.REC.1400.612). In this study, the participants were first asked to complete a written consent form. Then, a standardized questionnaire was used by trained experts in order to gather information about the participants’ demographic characteristics (age, sex, Body Mass Index (BMI), marital status, level of education, place of residence, occupational status, social and economic status, and family history of chronic diseases), sleep pattern, problems and underlying diseases (diabetes, cancer, Chronic Obstructive Pulmonary Disease (COPD), hypertension, blood levels of cholesterol, triglycerides, and High Density Lipoprotein (HDL), and other chronic diseases), and behavioral factors (smoking, alcohol consumption, hookah use, drug use, and physical activity) through interviews, laboratory experiments, and physical examination. Since Kherameh cohort was a part of the main project of the PERSIAN cohort, the validity and reliability of the questionnaire had been previously checked by the Persian cohort national team [19]. For the laboratory experiments, the individuals were requested to refrain from eating food and liquids, smoking, and consuming alcohol for 12 h prior to blood sampling. Weight was measured with light clothing and without shoes using a SECA scale (made in Germany), and height was measured using a standard measuring tape. BMI was also calculated by dividing body weight (kg) by height squared (m). Accordingly, the participants were divided into three categories; i.e., less than 25, 25–29, and > 30 kg/m^2^ [20]. Furthermore, blood levels of glucose, triglycerides, HDL, and cholesterol were measured using the MINDRAY brand tool (made in Japan) and Pars test kit. Dyslipidemia was defined as cholesterol levels above 200 mg/dL or triglyceride serum levels above 150 mg/dL. Additionally, diabetes was defined as a history of diabetes or fasting blood sugar above 126 mg/dL [21, 22]. The participants’ average physical activity was also evaluated during the past year. Moreover, their socioeconomic status was determined by considering such characteristics as home ownership, house size, number of indoor bathrooms, having a car, car price, domestic and international travels, and having a mobile phone, TV, vacuum cleaner, washing machine, refrigerator, microwave oven, and computer. Blood pressure was measured on the participants’ right arms after a five-minute rest in sitting position using a standard calibrated sphygmomanometer (Reister Model, Germany). Blood pressure was measured twice with a minimum interval of ten minutes and the mean was recorded. According to the European Hypertension Management Guidelines, hypertension was defined as systolic blood pressure greater than 140 mmHg and diastolic blood pressure greater than 90 mmHg [23].

### Inclusion criteria

The inclusion criteria of this study were the same as those considered in Kherameh cohort study (one of the eighteen cohorts under the name of Persian cohort in Iran). The first inclusion criterion was aging 40–70 years, because people's behaviors and lifestyles are largely established in this age range. Besides, these people are usually in the active period of their lives and have the ability to participate in the study. Finally, the events under investigation can more probably be seen in this age group. The second inclusion criterion of the study was living in Kherameh. The participants had to live in the study area for at least nine months, so that they were somewhat adapted to the environmental and cultural conditions.

### Exclusion criteria

The individuals with mental disorders including mental retardation and other untreated acute illnesses, those who were unwilling to participate in the study, and those who did not attend the designated clinics for examinations were excluded from the research.

### Patients with cardiovascular disease

The individuals with ischemic heart disease including heart failure, angina, and myocardial infarction who were previously diagnosed in Kherameh cohort were included in the present study.

### Statistical analysis

Normally distributed continuous variables were presented as mean ± Standard Deviation (SD), while non-normally distributed ones were presented as median (Q1-Q3). Additionally, qualitative variables were presented as number (percentage). The relationships between the categorical variables were assessed by chi-square test and the means were compared via independent t-test. Besides, logistic regression was used to model and predict the factors related to CVD. Moreover, the Age-Standardized Prevalence Rate (ASPR) of CVD was determined using the standard Asian population [24]. All statistical analyses were conducted by the SPSS 23 software and Stata 12 software. All p-values were two-tailed and the significance level was set at 0.05.

## Results

This study was performed on 10,663 individuals whose baseline information has been shown in Table 1. Among the participants, 5944 (55.7%) were female with the mean age of 52.2 ± 8.2 years and 4719 (44.3%) were male with the mean age of 52.5 ± 7.8 years. Among the patients with CVD, 83.9% were unmarried (widowed or divorced), 64.2% were illiterate, 62.4% lived in rural areas, and 60.9% were unemployed. Moreover, 58.3% of the patients had high blood pressure and 28.1% had DM. The results revealed a higher prevalence of CVD in the individuals who were overweight and obese (66.2%, *p* < 0.001), unmarried (83.9%,  * p* < 0.001), illiterate (64.2%, *p* < 0.001), and unemployed (60.9%, *p* < 0.001) as well as in those with a high economic status (49.9%, *p* = 0.049).

**Table 1:** Demographic and socioeconomic variables of the participants according to the CVD status in Kherameh cohort study

Variable	CVD		No (n = 9552) n (%)	*p* value
	All participants n (%) n = 10,663	Yes (n = 1111) n (%)		
Age (years)				
40–49	4639 (43.7)	312 (27.9)	4330 (45.6)	< 0.001
50–59	3759 (35.4)	442 (39.9)	3317 (34.9)	
60–70	2218 (20.9)	357 (32.2)	1861 (19.6)	
Sex				
Male	4716 (44.2)	481 (43.3)	4238 (44.4)	0.49
Female	5942 (55.7)	630 (56.7)	5314 (55.6)	
BMI (kg/m^2^)				
< 18.5	414 (3.9)	29 (2.6)	385 (4)	< 0.001
18.5–25	3883 (36.4)	347 (31.3)	3536 (37)	
25–29	4449 (41.7)	489 (44.1)	3960 (41.5)	
> 30	1913 (17.9)	245 (22.1)	1668 (17.5)	
Marital status				
Married	9492 (89)	179 (16.1)	8560 (89.6)	< 0.001
Unmarried	1171 (11)	932 (83.9)	992 (10.4)	
Education level				
Illiterate	5587 (52.4)	713 (64.2)	4874 (51)	< 0.001
Diploma and below	4514 (42.3)	373 (33.6)	4141 (43.4)	
Academic	562 (5.3)	25 (2.3)	537 (5.6)	
Living place				
Urban	4416 (41.4)	481 (43.3)	3935 (41.2)	0.179
Rural	6247 (58.6)	630 (56.7)	5617 (58.8)	
Employment				
No	5147 (48.3)	677 (60.9)	4470 (46.8)	< 0.001
Yes	5516 (51.7)	434 (39.1)	5082 (53.2)	
Physical activity				
Light	2670 (25)	426 (38.3)	2244 (23.5)	< 0.001
Moderate	2666 (25)	276 (24.8)	2390 (25)	
High	2664 (25)	224 (20.2)	2440 (25.5)	
Severe	2663 (25)	185 (16.7)	2478 (25.9)	
Socioeconomic status				
Low	2667 (25)	258 (23.2)	2409 (25.2)	0.049
Moderate	2977 (27.9)	299 (26.9)	2678 (28)	
High	5019 (47.1)	554 (49.9)	4465 (46.7)	
Unintentional naps				
No	5178 (48.6)	464 (41.8)	4714 (49.4)	< 0.001
Yes	5485 (51.4)	647 (58.2)	4838 (50.6)	
Use of sleeping pills				
No	9735 (91.3)	916 (82.4)	8819 (92.3)	< 0.001
Yes	928 (8.7)	195 (17.6)	733 (7.7)	

BMI, body mass index

The prevalence of different clinical and behavioral factors has been presented in Table 2. Among these factors, smoking (28.3%, *p* = 0.018), opium use (18.2%, *p* = 0.039), and high triglyceride levels (31.6%, *p* = 0.011) were more prevalent among the people with CVD. The prevalence of CVD was also higher among the individuals with high blood pressure (58.3%, *p* < 0.001) and DM (28.1%, *p* < 0.001).

**Table 2:** The clinical and behavioral variables of the participants according to the CVD status in Kherameh cohort

Variable	CVD		No (n = 9552) n (%)	*p* value
	All participants n (%) n = 10,663	Yes (n = 1111) n (%)		
Diabetes				
No	9070 (85.1)	799 (71.9)	8271 (86.6)	< 0.001
Yes	1593 (14.9)	312 (28.1)	1281 (13.4)	
Cancer				
No	10,609 (99.5)	1100 (99)	9509 (99.5)	< 0.001
Yes	54 (0.5)	11 (1)	43 (0.5)	
COPD				
No	10,369 (97.2)	1055 (95)	9314 (97.5)	< 0.001
Yes	294 (2.8)	56 (5)	238 (2.5)	
High blood pressure				
No	8153 (76.5)	463 (41.7)	7690 (80.5)	< 0.001
Yes	2510 (23.5)	648 (58.3)	1862 (19.5)	
High cholesterol level				
No	7014 (65.8)	772 (69.5)	6242 (65.3)	0.006
Yes	3649 (34.2)	339 (30.5)	3310 (34.7)	
High triglyceride level				
No	7637 (71.6)	760 (68.4)	6877 (72.1)	0.011
Yes	3018 (28.3)	351 (31.6)	2667 (27.9)	
HDL (mg/dl)				
< 40 for males and < 50 for females	4798 (45)	515 (46.4)	4283 (44.9)	0.329
≥ 40 for males and ≥ 50 for females	5860 (55)	595 (53.6)	5265 (55.1)	
White blood cell count (× 1000 cells/mm^3^)				
< 5.8	4189 (39.3)	390 (35.1)	3799 (39.8)	0.006
5.8–7	3129 (29.3)	337 (30.3)	2792 (29.3)	
> 7	3337 (31.3)	384 (34.6)	2953 (30.9)	
Opium use				
No	8949 (83.9)	908 (81.8)	8041 (84.2)	0.039
Yes	1710 (16)	202 (18.2)	1508 (15.8)	
Hookah use				
No	10,115 (94.9)	1062 (95.6)	9053 (94.8)	0.28
Yes	548 (5.1)	49 (4.4)	499 (5.2)	
Smoking				
No	7956 (74.6)	797 (71.7)	7163 (75)	0.018
Yes	2704 (25.4)	314 (28.3)	2389 (25)	
Alcohol consumption				
No	10,095 (94.7)	1066 (95.9)	9029 (94.5)	0.045
Median serum creatinine (mmol/l)	0.9 (0.9, 1)			< 0.001

COPD, chronic obstructive pulmonary disease; HDL, high density lipoprotein

**Fig. 2:**
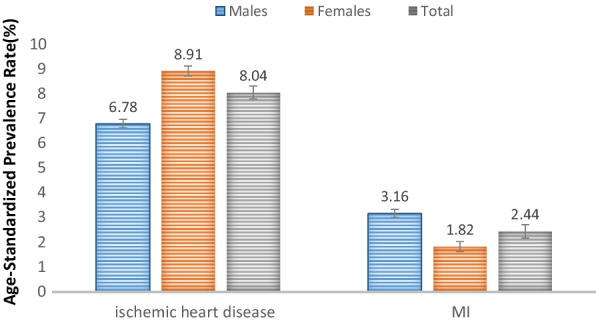
Age-standardized prevalence rate based on different types of CVD, adjusted to the standard Asian population. *Note*: The error bars represent the 95% CI

Based on the results presented in Table 3, the crude prevalence and ASPR of CVD were 10.4% (95% CI 9.8–11) and 10.3% (95% CI 10–10.6), respectively. Besides, the ASPR was 10.39 in males (95% CI 10.2–10.6%) and 10.21 in females (95% CI 9.9–10.4%). The ASPRs according to the types of CVD have been presented in Fig. 2.

**Table 3:** Crude and age-standardized prevalence rate of CVD according to sex in the adults aged 40–60 years in Kherameh cohort

	95% CI			95% CI		
	Crude	Upper	Lower	ASPR	Lower	Upper
Both sexes	10.41	9.8	11	10.3	10	10.6
Male	10.19	9.3	11	10.39	10.2	10.6
Female	10.6	9.8	11	10.21	9.9	10.4

ASPR, age-standardized prevalence rate

According to the results presented in Table 4, multivariate logistic regression analysis showed that the odds of having CVD was 2.25 times higher among the individuals aged 50–60 years compared to those aged 40–50 years, 1.66 times higher in opium users than in non-opium users, 1.37 times higher in smokers compared to non-smokers, 1.62 times higher amongst obese individuals (BMI > 30 kg/m^2^) in comparison to lean ones (BMI < 18.5 kg/m^2^), and 2.03 times higher in regular users of sleeping pills than in non-consumers. Considering the clinical variables, the odds of having CVD was 4.02 times higher in hypertensive individuals than in normotensive ones, 1.24 times higher in diabetics than in non-diabetics, and 1.71 times higher among the individuals with COPD compared to those without this disease.

**Table 4:** Predictive factors of CVD in Kherameh population according to multivariate logistic regression analysis

Variable	Crude OR (95% CI)	Adjusted OR (95% CI)
Age (years)		
40–50	1	1
50–60	1.86 (1.60, 2.17)*	1.63 (1.39, 1.91)*
60–70	2.68 (2.28, 3.16)*	2.02 (1.70, 2.40)*
Sex		
Male	1	
Female	1.04 (0.92,1.18)	
Body mass index (kg/m^2^)		
< 18.5	1	1
18.5–24.9	1.30 (0.87, 1.93)	1.25 (0.82, 1.90)
25–29.9	1.63 (1.11, 2.41)*	1.47 (0.96, 2.25)
≥ 30	1.95 (1.30, 2.91)*	1.62 (1.04, 2.52)*
Marital status		
Married	1	
Unmarried	1.65 (1.39, 1.97)*	
Education level		
Illiterate	1	1
Diploma and below	0.61 (0.54, 0.70)*	1.01 (0.86, 1.20)
Academic	0.31 (0.21, 0.47)*	0.56 (0.35, 0.88)*
Living place		
Urban	1	
Rural	0.92 (0.81,1.04)	
Employment		
No	1	
Yes	0.56 (0.49, 0.64)*	
Physical activity		
Light	1	1
Moderate	0.60 (0.51, 0.71)*	0.89 (0.74, 1.06)
High	0.48 (0.40, 0.57)*	0.8 (0.66, 0.97)*
Severe	0.39 (0.32, 0.47)*	0.73 (0.59, 0.90)*
Socioeconomic status		
Low	1	1
Moderate	1.04 (0.87, 1.24)	1.02 (0.84, 1.23)
High	1.15 (0.99, 1.35)	1.21 (1.01, 1.44)*
Unintentional naps		
No	1	1
Yes	1.35 (1.19,1.54)*	1.15 (1.005, 1.33)*
Use of sleeping pills		
No	1	1
Yes	2.56 (2.15, 3.04)*	2.03 (1.68, 2.46)*
Diabetes		
No	1	1
Yes	2.52 (2.18,2.91)*	1.24 (1.06, 1.46)*
Cancer		
No	1	
Yes	2.21 (1.13, 4.30)*	
Chronic obstructive pulmonary disease		
No	1	1
Yes	2.07 (1.54, 2.79)*	1.71 (1.23, 2.38)*
High blood pressure		
No	1	1
Yes	5.78 (5.07, 6.58)*	4.02 (3.46, 4.67)*
High triglyceride		
No	1	1
Yes	1.19 (1.04, 1.36)*	1.30 (1.11, 1.52)*
High cholesterol		
No	1	1
Yes	0.83 (0.72, 0.94)*	0.82 (0.71, 0.94)*
White blood cell count (× 1000 cells/mm^3^)		
< 5.8	1	
5.8–7	1.17 (1.08, 1.37)*	
> 7	1.26 (1.09, 1.47)*	
Median serum creatinine (mmol/l)	3.20 (2.33, 4.40)*	1.62 (1.14, 2.29)*
Opium use		
No	1	1
Yes	1.18 (1.09, 1.39)*	1.66 (1.35, 2.05)*
Hookah use		
No	1	
Yes	0.83 (0.62, 1.31)	
Alcohol use		
No	1	
Yes	0.72 (0.53, 0.99)*	
Smoking		
No	1	1
Yes	1.18 (1.02, 1.35)*	1.37 (1.13, 1.65)*

* Values represent *p* < 0.05

## Discussion

The present study aimed to estimate the prevalence and predictors of CVD among the people aged 40–70 years in Kherameh, southern Iran. The findings demonstrated that the prevalence of CVD was 10.3%. Among the demographic and socioeconomic variables, old age, high BMI (> 30 kg/m^2^), low level of education, low physical activity, high socioeconomic status, unintentional naps, and sleeping pill use were the risk factors for CVD. Among the clinical factors, history of diabetes, cancer, COPD, high blood pressure, and high creatinine level were the risk factors for CVD. Finally, among the behavioral variables, opium use and smoking were found to be risk factors, while hookah use and alcohol consumption had a protective role. However, only drug use and smoking were significant in multivariate analysis.

### Prevalence of CVD

The present study results showed that the prevalence of CVD was higher in south of Iran compared to other regions and countries. In this study, the prevalence of CVD was 10.3%, representing approximately 10,300 cases per 100,000 population. A study on the global burden of CVD also indicated that the standardized prevalence of CVD was more than 9,000 cases per 100,000 population in Iran as well as in Morocco, Oman, Zambia, and West Africa. In contrast, the standard prevalence of CVD was less than 5,000 per 100,000 population in Singapore, Japan, South Korea, Italy, Western Europe, and the United States, which was lower compared to the estimated prevalence in the current investigation [25]. This discrepancy might be due to the complex interaction of various environmental and genetic factors that play an important role in the development of CVD as well as to the differences in the prevalence of cardio metabolic risk factors (e.g., hyperglycemia, hypertension, dyslipidemia, and central obesity) [26]. The prevalence of these risk factors has been found to increase in Iran in recent years [27]. However, the present study results revealed no significant difference between males and females concerning the prevalence of CVD. In contrast, other studies reported that males were at a higher risk of CVD development [13, 18]. On the other hand, Aapelman et al. reported a higher prevalence of CVD in females than in males [28]. In another study, Zeidan et al. disclosed that significant differences between the two sexes disappeared with age [17]. These contradictory results might be associated with different age groups and different types of CVD. In addition, some studies have attributed the differences between males and females in terms of CVD to pregnancy, menopausal age, and hormonal changes [28–32].The lack of a significant difference between the two sexes regarding the prevalence of CVD in the present study might be associated with the fact that the participants were within the age range of 40–70 years. In fact, the females were at the menopause age and, consequently, had less supportive physiological factors (estrogen and progesterone). Evidence has shown that the incidence of CVD is higher in postmenopausal women than in premenopausal ones and that estrogen supplementation does not reduce the risk of CVD in postmenopausal women [33–35].

### The association between the demographic and socioeconomic variables and CVD

Age is a known risk factor for CVD. The present study findings revealed an increase in the odds of CVD development in older adults. Previous studies also showed that age played a vital role in the deterioration of cardiovascular function, thereby enhancing the risk of CVD in the elderly population [36, 37].

In the current research, a positive association was found between CVD and higher BMI, which was consistent with the findings of other studies [38]. Obesity has been found to exert numerous adverse effects on cardiovascular function and structure, increase the risk of CVD, and increase the risk factors associated with CVD such as hypertension, diabetes, insulin resistance, and sleep apnea syndrome [39–41].

In the present investigation, the individuals with low education levels seemed to be more likely to develop CVD. Other studies have also suggested an inverse association between CVD and education level [42–44].

In the current study, socioeconomic status remained in the multivariate analysis after adjusting for other risk factors. Based on the results, the people with a high socioeconomic status were more likely to develop CVD. However, conflicting results have been obtained in other studies regarding the relationship between the socioeconomic status and CVD [45]. Some studies performed in developed countries revealed an inverse relationship between the socioeconomic status and CVD, while those carried out in developing countries showed a direct relationship between the two variables [42, 46, 47]. The direct correlation may be attributed to the fact that rich people in developing countries such as Iran have higher behavioral risk factors such as poor diet and high rates of smoking compared to poor people, as other studies have noted [48, 49].Previous studies demonstrated that short and long sleep durations were associated with an increased risk of CVD [50–53]. Consistently, the present study findings indicated that unintentional nap was a risk factor for CVD and taking sleeping pills increased the odds of CVD development. Some physicians attribute insomnia to the need for sleeping pills for underlying medical disorders such as CVD, high blood pressure, diabetes, and cancer [52, 54]. Therefore, the present study participants might suffer from insomnia due to CVD and, as a result, they were more likely to turn to sleeping pills. Yet, the relationship observed in cross-sectional studies should be interpreted with caution due to the weakness in showing the temporality [55].

### The association between the clinical and behavioral variables and CVD

Up to now, many studies have emphasized that high blood pressure, diabetes, and COPD played an important role in the development of CVD [56–58]. In the same line, the current study showed a strong association between these variables and CVD. In this regard, blood pressure, diabetes, and COPD were the most important risk factors, which was in agreement with the findings of other studies. Several prospective cohort studies have demonstrated hypertension as a strong risk factor for CVD [57, 59–61]. Additionally, Coronary Heart Disease (CHD) has been mentioned as the most common early manifestation of CVD in patients with DM [56]. Moreover, the results of a meta-analysis on observational studies indicated that COPD increased the risk of CVD by 2.5 times, which was consistent with the results of the present investigation [58]. However, some other observational studies showed that COPD was more common in the people presented with various forms of CVD [62, 63]. Despite these contradictory results, evidence based on studies, especially longitudinal investigations and meta-analyses, has been in favor of the increased risk of CVD in patients with COPD. Curkendall et al., for example, estimated the age-adjusted risk ratio of heart failure as 4.5 among the individuals with COPD in a Canadian cohort [64–67].

The findings of the present research showed an inverse relationship between the total cholesterol level and CVD, which was on the contrary to the findings of the study carried out by Ghaemian and Bangalore [13, 68]. The observed inverse relationship might result from the practitioners’ recommendations for diet change and use of medications.

In the present study, creatinine and triglyceride levels were significantly associated with CVD, which was consistent with the results of other studies conducted on the issue. For instance, a meta-analysis demonstrated that triglyceride level was an independent risk factor for CHD, even after adjustment for HDL level [69]. Interpretation of the association between creatinine level and CVD should be done with caution, because creatinine levels increase in patients with kidney disease and CVD decreases renal function [70]. Although a number of recent epidemiological studies have revealed chronic kidney disease as an independent risk factor for CVD, this relationship may be due to the presence of kidney disease. However, the information of patients suffering from kidney disease was not available here for adjustment [71, 72].

Among the behavioral factors in the current study, opium use and smoking were found to be risk factors, while hookah use and alcohol consumption had a protective role. However, the results of multivariate analysis only showed drug use and cigarette smoking to be significant. In the same vein, Khalili et al. reported that based on dose–response and duration of use, opium was directly associated with an increased risk of CVD [73]. Nonetheless, conflicting results have been obtained regarding the relationship between opium use and CVD. Niaki et al. disclosed that opium use increased the odds of CVD by 24.5 times [74]. Sadeghian et al. also stated that opium use was a major risk factor for ischemic heart disease. However, Sabzi et al. and Marmor et al. maintained that opium use was a protective factor for atrial fibrillation (adjusted odds ratio = 0.37) and coronary artery disease (adjusted odds ratio = 0.43) [75, 76]. On the other hand, in the case–control study performed by Azimzadeh et al. and the cross-sectional study done by Hosseini et al., no significant association was observed between opium use and CVD [77, 78]. The differences among the results of the abovementioned studies might be attributed to the following reasons. Firstly, the possible confounding role of smoking might have not been considered in all the studies [79]. Secondly, the dose of opium consumption, method of use, and duration of use might vary in different studies [80]. Thirdly, in some areas, especially in Iran, opium is not a pure substance and contains impurities such as lead, which may increase the risk of CVD amongst opium users [44–46]. Fourthly, considering the social stigma associated with addiction, people might have hidden their addiction, which could have led to information bias [81, 82].

Similar to other studies, the present research revealed smoking as a risk factor for CVD. Hackshaw et al. also conducted a meta-analysis and reported an association between smoking and CHD [83]. In the same line, previous studies demonstrated a relationship between tobacco smoking and the physiological, pathological, and metabolic factors contributing to the atherosclerotic process and other mechanisms resulting in CVD-related morbidity and mortality [84–86].

### Limitation

This study had a few limitations. Firstly, the cross-sectional design of the study made it difficult to rule out the inverse causation of variables, especially for cholesterol level, hookah use, and alcohol consumption. Secondly, due to the stigma associated with drug use and alcohol consumption in Iran, the participants might have concealed these behaviors, which could have biased the observed relationships, Thirdly, information regarding genetic, racial, and ethnic backgrounds was not available to determine their roles. Despite these limitations, a strong point of the study was its large sample size of males and females, which was the representative of the population of Kherameh. In addition, highly accurate cohort study data were used in this study.

## Conclusion

The prevalence of CVD was found to be relatively higher in Kherameh (southern Iran) compared to other places. In addition, old age, obesity, taking sleeping pills, hypertension, drug use, and COPD had the highest odds ratios. Therefore, plans are recommended to be made in order to reduce the modifiable risk factors. Furthermore, contrary to the existing misconceptions about the positive effects of opium on heart diseases, opium was found to be a risk factor in this research. Thus, the public should be informed about the dangerous effects of opium use [79]. Moreover, further longitudinal cohort studies are recommended to be performed focusing on the incidence data of the related factors, especially sleeping pill use. More attention should also be paid to the individuals who have risk factors related to CVD, especially those who use opium and sleeping pills. Future studies are also needed to improve our understanding of the complex risk factors underlying the development of CVD in females.

## Data Availability

The datasets generated and analyzed during the current study are not publicly available due to data are not public, but are available from the corresponding author on reasonable request.

## Abbreviations

CVD: Cardiovascular disease
ASPR: Age-standardized prevalence rate
FBS: Fasting blood sugar
TC: Total cholesterol
HDL-C: High-density lipoprotein-cholesterol
LDL-C: Low-density lipoprotein-cholesterol
TG: Triglyceride
BMI: Body mass index
COPD: Chronic obstructive pulmonary disease
